# Implementation of Single-Pill Combination Medication for Hypertension Treatment by Nonphysician Health Care Workers at Primary Healthcare Facilities in Nigeria: An Explanatory Mixed Methods Study

**DOI:** 10.5334/gh.1507

**Published:** 2025-12-22

**Authors:** Emmanuel I. Okpetu, Chisom Obiezu-Umeh, Boni M. Ale, Abigail S. Baldridge, Rosemary C. B. Okoli, Grace J. Shedul, Gabriel L. Shedul, Nanna R. Ripiye, Ikechukwu A. Orji, Lisa R. Hirschhorn, Dike B. Ojji, Mark D. Huffman, on behalf of the Hypertension Treatment in Nigeria Program Investigators

**Affiliations:** 1 Department of Medical Social Sciences and Health Science Integrated Program (Health Services and Outcomes Research) Northwestern University Feinberg School of Medicine Chicago, Illinois, USA; 2 Cardiovascular Research Unit, University of Abuja Teaching Hospital, Nigeria; 3 Department of Medical Social Sciences Feinberg School of Medicine, Northwestern University, Chicago, Illinois, USA; 4 Holo Global Health Research Institute, Nairobi, Kenya; 5 Department of Preventive Medicine, Northwestern University Feinberg School of Medicine, USA; 6 School of Integrated Sciences, Sustainability, and Public Health, College of Health, Science, and Technology, University of Illinois, Springfield, USA; 7 Robert J Havey Institute for Global Health, Feinberg School of Medicine, Northwestern University, Chicago, Illinois, USA; 8 Department of Internal Medicine, Faculty of Clinical Sciences University of Abuja, Abuja, Nigeria; 9 Division of Cardiology and Global Health Center, Washington University in St. Louis, St. Louis, USA; 10 School of Public Health, Washington University in St. Louis, St. Louis, USA; 11 The George Institute for Global Health, UNSW, Sydney, Australia

**Keywords:** hypertension, fixed-dose/single-pill combination, nonphysician healthcare worker, community healthcare worker, primary healthcare, task shifting/sharing, Nigeria

## Abstract

**Background:**

Single-pill combination (SPC) therapy improves hypertension control; however, its implementation in primary care settings remains limited. In Nigeria, there is insufficient evidence on factors influencing SPC uptake, particularly from the perspective of healthcare workers (HCWs). This study examined the implementation of SPC medications for hypertension treatment by nonphysician HCWs at primary healthcare facilities (PHCs) in Nigeria.

**Methods:**

An explanatory sequential mixed methods study was conducted, building on a cluster randomized controlled trial embedded within the Hypertension Treatment in Nigeria Program. The trial compared SPC medications with free-equivalent combination therapies across 60 PHCs (January–June 2021). A subsequent qualitative component (September–December 2021) included two focus group discussions from 30 PHCs assigned to the SPC arm of the trial and five key informant interviews. The Reach, Effectiveness, Adoption, Implementation, Maintenance (RE-AIM) framework was used to assess implementation outcomes and identify facilitators and barriers. Integration of quantitative and qualitative findings was guided by the RE-AIM Qualitative Evaluation for Systematic Translation framework (QuEST).

**Results:**

All 30 PHCs assigned to dispense SPCs adopted the medications (Reach/Adoption). Effectiveness: Blood pressure control (<140/90 mm Hg) was 54% (95% CI: 0.52, 0.56) in the SPC arm and 48% (95% CI: 0.46, 0.50) in the free-equivalent arm (cluster-adjusted p = 0.29). Monthly SPC use ranged from 21–37% across sites (Implementation), and 49% of patients remained in care at six months (Maintenance). Facilitators included training on SPC protocols, simplicity of dispensing the regimen, and perceived improvements in patient adherence. Challenges included SPC stockouts and concerns regarding nonphysician HCW capacity to manage complex cases. Policymakers identified the potential role of a Drug Revolving Fund (DRF) to support sustained SPC supply.

**Conclusions:**

The findings indicate favorable implementation outcomes associated with SPC use by nonphysician HCWs in PHCs. Addressing supply challenges, maintaining training, and providing supportive supervision may be important for sustaining SPC-based hypertension treatment.

## Introduction

In Nigeria, the burden of elevated blood pressure (BP) or hypertension is high with a prevalence of 38%, yet treatment (34%) and control (12%) are low (1). Multiple factors contribute to suboptimal BP control, including patient non-adherence to prescribed medications, treatment inertia, limited healthcare worker (HCW) capacity for appropriate prescription and counseling, high salt intake, and socioeconomic barriers (2,3,4). Single-pill combination (SPC) medications improve treatment adherence, which leads to better BP control (5,6). SPCs make service delivery and prescription practices less complicated for HCWs in the treatment of hypertension, facilitate supervision, and enhance standardization of hypertension care, with simplified protocols (5,6,7,8,9). Evidence from previous trials demonstrates that SPCs are at least as effective as, and in some cases superior to, free-equivalent combinations (10,11,12). Considering the importance of SPCs in the control of BP, the World Health Organization (WHO) recommended the inclusion of SPC antihypertensives in essential medicines lists (6,8). SPC antihypertensives have also been recommended in Nigeria’s clinical guidelines (13). However, there is a paucity of studies on HCW perspectives on the use of SPCs, especially in Nigeria and low- and middle-income countries (LMICs) where the burden of hypertension is high (14). Nonphysician HCWs in Nigeria have prior experience using SPCs in the management of HIV and tuberculosis, supported by task-sharing initiatives and enabling policies of the Federal Ministry of Health (15,16,17,18). These transferable skills in the use of SPC medications for infectious diseases have been leveraged and strengthened by the Hypertension Treatment in Nigeria (HTN) Program (NCT04158154), leading to the training and provision of a guideline on the use of SPC for community health extension workers (CHEWs) and other nonphysician HCWs in primary care settings (19,20,21) who were not previously licensed to prescribe hypertension medication in Nigeria (22,23). While the parent HTN Program and related publications use the term ‘fixed-dose combination’ (FDC), the term ‘SPC’ is adopted primarily in this study to align with the 2021 WHO hypertension guideline, as both terms refer to the co-formulation of two or more antihypertensive agents in a single tablet (19,21,24,25).

The HTN Program included an embedded cluster randomized controlled trial (RCT) comparing SPC medications with free-equivalent combination therapies (19). The HTN Program used a type II hybrid implementation-effectiveness, interrupted time series design to evaluate the effect of a multilevel bundle based on the WHO’s HEARTS technical package (21,26). The Nigerian national hypertension treatment protocol used in the HTN Program includes four steps based on the measured BP and previous treatment, including a single medication at Step 1 (amlodipine), two medications at Steps 2 and 3 (amlodipine, losartan), and three medications at Step 4 (amlodipine, losartan, hydrochlorothiazide) (19). The medications in Steps 2 and 3 could be prescribed as free-equivalent or as an SPC, based on site allocation (19).

The cluster RCT found SPCs to be as effective as the free-equivalent hypertensive medications within the HTN Program (19). Given this comparable effectiveness, alongside the additional advantages of improved patient adherence and simplified prescribing for HCWs, the findings support the need for optimization and scale-up of SPC use in similar settings. To inform strategies for scaling and sustaining SPC use by nonphysician HCWs in Nigeria, this study employed an explanatory sequential mixed methods design (27). Quantitative findings from the embedded RCT informed the qualitative inquiry, which used the RE-AIM (Reach, Effectiveness, Adoption, Implementation, Maintenance) QuEST (Qualitative Evaluation for Systematic Translation) framework to explain and explore how contextual factors influence implementation and effectiveness outcomes (27,28). ‘Reach’ refers to the proportion of facilities engaged in the intervention. ‘Effectiveness’ reflects the impact of SPCs on BP control. ‘Adoption’ represents the proportion of primary healthcare facilities (PHCs) initiating SPC use. ‘Implementation’ assesses fidelity to SPC prescribing. ‘Maintenance’ considers the sustained delivery of SPCs and follow-up practices over time as beyond initial implementation. Definitions of implementation outcomes and qualitative explanations are summarized in Table 1.

**Table 1:** Definitions of implementation outcomes for the use of single-pill combination therapy (SPC) in the Hypertension Treatment in Nigeria (HTN) Program and associated quantitative and qualitative data using the RE-AIM QuEST framework.


RE-AIM DOMAIN	MEASURES/INDICATOR	QUANTITATIVE DATASOURCE: HTN PROGRAM	QUALITATIVE DATASOURCE: FGDS AND KIIS

Reach	Intervention: Proportion of primary healthcare facilities (PHCs) selected to participate in implementing SPC that accepted to dispense SPC	Proportion of targeted PHCs that agreed to be assigned to dispense SPC	Reasons for facility participation in SPC prescribing Factors influence patients’ access to treatment with SPC

Effectiveness	Effectiveness of the use of SPC by trained nonphysician HCWs in BP control	BP control rates in SPC-assigned PHCs	Perception/beliefs about effectiveness of SPC

Adoption	HCWs dispensing SPC in assigned PHCs	Proportion of SPC-assigned PHCs that dispensed any SPC	Factors contributing to the nonphysician PHC HCWs dispensing SPC Perception/opinions and barriers/facilitators to successful adoption Role of strategies in supporting adoption

	Strategy: Training of nonphysician HCWs on the use of a protocol-based prescription of SPC	Proportion of intervention PHCs with at least one nonphysician HCW trained on the use of SPC	Relevance of training Training needs and gaps Training approaches

Implementation (fidelity)	Consistency with dispensing SPC according to protocols by HCWs	Proportion of enrolled patients in SPC-assigned PHCs who were eligible for Step 2 or 3 who received prescriptions of SPC each month	Factors contributing to the PHC nonphysician HCWs adhering to SPC protocols Barriers/facilitators to medication adherence

Maintenance	Continuity and long-term dispensing of SPC therapy by HCWs	Proportion of patients started on SPC who followed up on SPC within the six-month trial (patients seen and prescribed with SPC at least twice within the six months as defined in the HTN Program)	Factors contributing to the PHC nonphysician HCWs maintaining use of SPC Barriers/facilitators to maintenance of SPC use by patients (according to the nonphysician HCWs) Recommendations on sustaining SPC therapy at the facility or patient levels

	BP control maintained above baseline in SPC assigned PHCs		


## Methods

### Study design

This explanatory sequential mixed methods study drew on quantitative data from a cluster RCT embedded within the HTN Program (27). Details of the HTN Program have been previously published (20,21). Briefly, 60 PHCs from six area councils (local governments) in the Federal Capital Territory of Nigeria were selected for the HTN Program, which conducted an embedded cluster RCT (1:1, n = 60 clusters) from January 2021 to June 2021 to evaluate the effectiveness and implementation of a two-drug SPC at Steps 2 and 3 provided to patients, compared with providing separate pills, on the odds of hypertension control at six months (19). SPC antihypertensive therapy was implemented in 30 of the 60 participating PHCs. Nonphysician HCWs, including CHEWs and nurses, received structured training on a simplified, protocol-based hypertension treatment algorithm covering screening, diagnosis, patient education, and SPC use at Steps 2 and 3. Interactive sessions included demonstrations and role-playing. During the trial, SPC medications were provided at no cost; a Drug Revolving Fund (DRF) scheme subsidized costs afterward. Eligible patients identified through facility-based screening were enrolled, initiated on SPC therapy, and followed up monthly for BP monitoring, medication refills, and documentation. To maintain fidelity to the treatment protocol, nonphysician HCWs received ongoing on-site mentorship and quarterly supportive supervision from the research team (19).

Quantitative data on RE-AIM outcomes were used to inform the subsequent qualitative data collection, which was conducted between September and December 2021 using focus group discussions (FGDs) and key informant interviews (KIIs). The Consolidated criteria for Reporting Qualitative research (COREQ) checklist guided the reporting of qualitative methods and findings (Supplement 1) (29). The results of the embedded cluster RCT have been published (19) while relevant program reports were reported as proportions.

### Quantitative study

#### Quantitative Data Collection

Nonphysician HCWs collected patient data at baseline and during monthly follow-up visits over six months. Standardized forms adapted from the WHO HEARTS technical package were used to record demographics, BP readings (average of two measurements taken five minutes apart using validated electronic devices), medication prescriptions, and retention in care. Data was entered electronically into a central database and reviewed weekly for quality assurance and completeness (21).

#### Quantitative Outcomes

Quantitative data on the use of SPC for the treatment of hypertension in the HTN Program were derived from the published results of the embedded trial (19) and relevant programmatic reports of the HTN Program. The trial established the clinical effectiveness of SPC therapy, with a mean BP control rate of 54% among 2,381 patients at six months (19). These findings provide the foundation for this study’s focus on evaluating implementation outcomes, specifically Reach, Adoption, Implementation fidelity, and Maintenance.

### Qualitative study

#### Sampling and Recruitment

Participants for the two FGDs were purposively selected from the 30 PHCs randomized to dispense SPC medications in the embedded RCT. One FGD included CHEWs, and the other involved nurses, with representation from all six participating area councils. The purposive sampling aimed to ensure representation across PHCs and cadres of nonphysician HCWs involved in hypertension care. Each FGD targeted 12 participants, ensuring a representation of male and female HCWs, and inclusion of staff from PHCs with both the highest and lowest patient enrolment in the SPC arm. KIIs targeted five purposively selected stakeholders including policymakers, regulators, and healthcare professionals such as pharmacists, cardiologists, community health officers, nurses, and public health specialists involved in hypertension care, medication regulation, or oversight of nonphysician practices in primary care. Participants did not have the opportunity to review or revise their responses, and no repeat interviews were conducted. The sample size was determined based on thematic data saturation, defined as the point at which no new codes or insights emerged during analysis.

#### Development of Interview and Discussion Guides

Semi-structured guides for HCWs and key informants (Supplements 2 and 3) were developed using RE-AIM domains to explore implementation outcomes. Questions focused on experiences, barriers, facilitators, and perspectives on sustaining SPC use by nonphysician HCWs in PHCs. The guides were designed to complement and explain findings from the embedded RCT. Pretest of the focus group and interview guide conducted with a small group ensured clarity and contextual relevance.

#### Qualitative Data Collection

FGD and KII participants were invited via phone calls and formal letters, with participation arranged at mutually agreed, convenient locations. Participation was voluntary, and all participants provided written informed consent before data collection. Transportation costs were reimbursed through honoraria, as applicable. All FGDs and KIIs were conducted by the lead author (EIO), a public health physician trained in qualitative research and familiar with the primary healthcare context in which the study was conducted. FGD sessions were supported by assistants with public health background and experience conducting qualitative research. Both FGDs and KIIs were audio-recorded, de-identified, and transcribed verbatim by a professional transcriber. FGDs lasted an average of 45 minutes and KIIs averaged 30 minutes. To support reflexivity and reduce potential bias, the interviewer kept brief notes during data collection to document positionality and key decisions relevant to the analysis. Data saturation was reached when review of all transcripts yielded no new codes or themes.

#### Qualitative Data Analysis

Quantitative findings of the implementation outcomes from the original SPC trial informed the development of the semi-structured discussion and interview guides, with open-ended questions designed to explore contextual reasons for variation using the RE-AIM QuEST framework, which integrates qualitative inquiry into the framework to explain implementation barriers and contextual drivers of translation across settings (28). Guided by the RE-AIM QuEST framework, direct content analysis was employed while an inductive approach was also applied to elicit emergent themes directly from the data. Two trained qualitative researchers independently double-coded all transcripts using Dedoose software (v9.2.22, Los Angeles, CA: Sociocultural Research Consultants, LLC) (30). Coding discrepancies were resolved through iterative discussions until consensus was reached. Following completion of coding, the code report was generated and transferred into matrix displays in Microsoft Excel, where findings were categorized into themes aligned with RE-AIM dimensions. A joint-display matrix was then used to align quantitative RE-AIM outcomes with corresponding qualitative data on facilitators and barriers, providing an explanatory mixed methods interpretation of the findings.

### Mixed method data integration

Mixed methods analysis was performed to assess factors that facilitated or hindered the implementation and effectiveness of SPC. Guided by RE-AIM QuEST, qualitative data explained quantitative findings from the embedded trial and identified contextual factors influencing reach, adoption, fidelity, and maintenance. Additionally, findings from the quantitative data informed the development of the semi-structured guides.

## Results

### Quantitative results

There was wide reach, as all 30 PHCs assigned to provide SPC therapy for BP treatment implemented the intervention. Effectiveness was demonstrated by a mean BP rate of 54% (95% CI: 0.52, 0.56) among 2,381 patients at six months in the SPC arm and 48% (95% CI: 0.46, 0.50) among 2,046 in the free-equivalent arm (unadjusted risk difference = 6.0 pp; 95% CI: 2% to 10%, cluster adjusted p value = 0.29). Adoption was high, with all facilities dispensing SPC at least once during the study period. At least one nonphysician HCW in each PHC was trained to prescribe SPC in line with Steps 2 and 3 of the treatment protocol. Implementation fidelity improved progressively over the six-month trial, with the proportion of enrolled hypertensive patients who were dispensed an SPC at intervention sites increasing from 21% in Month 1 to 37% in Month 6. Maintenance at the patient level was moderate, with 49% of patients in the SPC arm remaining in care at six months. At baseline, none of the participating PHCs were dispensing SPCs; by six months, all SPC facilities were actively prescribing and dispensing the medication, a measure of facility-level maintenance, consistent with the protocol-defined indicators for maintenance of the intervention. At the facility-level, maintenance was also demonstrated by sustained treatment delivery above baseline values, with average facility-level BP control increasing from 14% at baseline to 54% at six months (19,21).

### Qualitative results

#### Characteristics of Study Participants

Characteristics of participants are summarized in Table 2. There were two FGDs with 24 participants in total. The first FGD included CHEWs: n = 12, 58% female, median age 41.5 years (range: 34–54), median work experience 14 years (range: 12–30), selected from among the 30 PHCs assigned to the SPC arm of the RCT. Participants included at least one from PHCs with the highest and lowest patient enrollment in the SPC arm. The second FGD included nurses: n = 12, 67% female, median age 52.5 years (range: 40–58), median work experience 18 years (range: 14–30), selected from SPC PHCs not represented in the CHEW group. In addition, five KIIs were conducted with stakeholders: 40% female, median age 50 years (range: 45–59), median professional experience 25 years (range: 15–33), representing regulatory bodies, policymakers, service delivery organizations, and professional associations involved in primary care and nonphysician practice in Nigeria.

**Table 2:** Characteristics of qualitative study participants.


QUALITATIVE DATA SOURCE	NUMBER OF PARTICIPANTS AND SEX DISTRIBUTION	PROFESSIONAL CADRE/ROLE	AGE DISTRIBUTION (YEARS)	YEARS OF PROFESSIONAL EXPERIENCE	ORGANIZATIONAL AFFILIATIONS

Focused Group Discussion 1	12 (7 females, 5 males)	12 Community health extension workers (CHEWs)	Median: 41.5 Range: 34–54	Median: 14 Range: 12–30	2 participants per area council

Focused Group Discussion 2	12 (8 females, 4 males)	12 Nurses	Median: 52.5 Range: 40–58	Median: 18 Range: 14–30	2 participants per area council

*Key Informant Interviews	5 (2 females, 3 males)	Physicians: 2 (1 cardiologist, 1 public health specialist) Pharmacist: 1 Policymaker: 4 Nurse: 1 ^Community health officer: 1	Median: 50 Range: 45–59	Median: 25 Range: 15–33	Primary Health Care Agency: 1 Ministry of Health: 1 Secondary health care facility: 1 Health worker regulatory bodies: 2 Professional associations: 2


*Some key informants had multiple roles or organizations so total number of roles and organizational affiliations was more than 5. ^Community health officer (CHO) is a CHEW that has an additional one year training to gain clinical and managerial skills essential for managing a PHC as an officer in charge.

#### Qualitative results

Themes were organized using the RE-AIM QuEST framework, with a focus on identifying key facilitators and barriers that influenced implementation outcomes. A summary of the thematic findings is presented in Table 3.

**Table 3:** Implementation outcomes of single-pill combination therapy implementation in the HTN program using the RE-AIM QuEST framework highlighting the quantitative and qualitative results.


OUTCOME	MEASURES/INDICATOR	QUANTITATIVE RESULTS	QUALITATIVE RESULTS	

			FACILITATORS	BARRIERS

**Reach**	Proportion of targeted primary healthcare facilities (PHCs) that agreed to be assigned to dispense SPC^	100% (30/30)	Availability of SPC medication at the PHCsFree SPC provision helped alleviate financial barriers and encouraged greater patient engagement	

**Effectiveness**	BP control rates in SPC-assigned PHCs**	54%	HCW perception of good BP control due to improved adherence from patients and tolerable side effects	

**Adoption**	Proportion of SPC-assigned PHCs that dispensed any SPC^	100% (30/30)	HCWs welcomed the use of SPC and advocated for expanded roles in hypertension care.Ease of use and reduced pill burden of SPCs facilitated uptake	Policymakers’ concerns about nonphysicians managing more than two-drug combinations and recommend restricting their role to early hypertension stages.

	Proportion of intervention PHCs with at least one nonphysician healthcare worker trained on the use of SPC^++^	100% (30/30)	Improved HCW capacity and confidence due to the provision of standardized treatment protocols and training on SPC	

**Implementation (fidelity)**	**HCW fidelity to prescribing SPC to eligible patients:** Proportion of patients in SPC-assigned PHCs (n = 30) who were eligible and received SPC (Step 2 or 3) each month over a six-month period^	**Month 1**: 21% (225/2,946) **Month 2**: 23% (241/2,862) **Month 3**: 29% (367/3,164) **Month 4:** 31% (337/3,080) **Month 5**: 34% (463/3,506) **Month 6:** 37% (540/3,928)	Adequate supervision and technical support facilitated adherence to protocols.	Occasional SPC stockouts, which disrupted consistent prescribing. Non-availability of multiple SPC options to cater to side effects and patient variability. Difficulty identifying which SPC component caused side effects.

**Maintenance**	Proportion of patients started on SPC who remained on SPC at the end of the 6-month cluster RCT (patients seen and prescribed with SPC at least twice within six months)	49% (1,154/2,381)	Patients’ satisfaction and enthusiasm for SPC therapy motivated regular attendance and adherence. Potential of a drug revolving fund to sustain the supply of SPC.	Concerns that some patients may be unable to afford SPC when fee-based model is introduced. Stockouts disrupt continuity of care.

	BP control maintained above baseline in assigned PHCs	14% at baseline vs 54% at 6 months		


**^** Sanuade OA, Ale BM, Baldridge AS, Orji IA, Shedul GL, Ojo TM, et al. Fixed-dose combination therapy-based protocol compared with free pill combination protocol: results of a cluster randomized trial. *The Journal of Clinical Hypertension*. 2023;25(2):127–136. DOI: https://doi.org/10.1111/jch.14632. **++** HTN Program reports on the training of HCWs. **Blood pressure control at the last visit among patients who had visited a PHC twice during the study period.

### Reach

#### Improved Access and Engagement in Hypertension Care

HCWs reported that the availability of SPC medication at the PHCs led to increased patient turnout for hypertension care, as reflected in higher attendance. Respondents also noted that providing the SPC for free helped alleviate financial barriers and encouraged greater patient engagement in the HTN Program. The provision of free medication thus functioned as a contextual facilitator that enhanced Reach during implementation:

Few people that used to come, we used to administer some drugs to them, but if we reached our limit, we referred them to our secondary health facility, which is the general hospital. But now with these drugs, many people are coming, even from neighboring villages, to access their drugs. (Nurse, PHC Facility Service Provider)

In my facility, many turn up because of these free drugs (SPCs). Some didn’t even know they were hypertensive until we screened them, and now they are happy to enroll and take their medications as required. (Nurse, PHC Facility Service Provider)

### Effectiveness

#### Perceived Effectiveness of SPC Therapy

HCWs highlighted the effectiveness of SPC therapy in achieving better BP control. Improved medication adherence was cited as a key factor, with respondents noting that patients on SPCs experienced significant BP reductions and reported minimal side effects:

The use of fixed-dose combination in hypertension treatment has gone a long way to achieving good BP control because you have better drug compliance, and when compliance is good, control levels improve. (Cardiologist, Secondary Healthcare Hospital)

Because it has really brought BP down to normal, and those placed on it are seeing good results with very few side effects. It seems to be well tolerated. (CHEW, PHC Facility Service Provider)

### Adoption

#### Improved HCW Capacity and Confidence

HCWs reported increased capacity to manage hypertension and dispense SPC medication effectively due to the provision of standardized treatment protocols and training. The participants further described that these resources enhanced their knowledge and confidence, enabling them to treat patients appropriately at the primary care level, as well as prescribe SPCs. Nonphysician HCWs also emphasized the critical role of training in clarifying their scope of practice and improving decision-making around patient management which includes medication options and combinations, and timely referrals when care exceeded their scope:

But now the treatment protocol has been given to us, guiding us on how we should manage the patients with hypertension and counsel them on adhering to their medication. Before, we mostly used medicines like Moduretic (Hydrochlorothiazide + Amiloride) and Aldomet (Methyldopa), but now we can use the different combination drugs. (CHEW, PHC Facility Service Provider)

Many health workers who didn’t know much about hypertension now know how to treat hypertension, even without referring them. (CHEW, PHC Facility Service Provider)

Training is the bedrock of a lot of things. If they are trained, they will know where their capacity starts, where it ends, where they can no longer handle and they would be, they should be able to know when to refer someone from their own little primary health care or facility. (Pharmacist policy maker, Federal Ministry of Health)

#### Support for Task-Sharing and Expanded Roles for Nonphysician HCWs

CHEWs and a key informant advocated for expanded responsibilities in managing hypertension and for greater recognition in national regulations for their practices:

If policies give more tasks to community health practitioners, it will help address high BP in remote communities and areas without general hospitals. (Regulatory Official for CHEWs)

Some key informants, however, recommended limiting nonphysician HCWs’ roles to early stages of hypertension management including screening, initiating treatment with SPCs, and referring patients when necessary:

Nonphysician HCWs should handle fixed-dose combinations of two agents first. If the patient isn’t controlled, they should be referred to a physician. (Cardiologist, Secondary Healthcare Hospital)

Yeah, yeah, they should have a role because we say primary health care is your first point of call, so if it’s your first point of call, you should be able to at least have an idea, check the person, you know screen, you should be able to recognize if the blood pressure is high or low and all that, and then do the referral which is, you know, what is expected. (Public health physician, Policy Maker, Federal Agency)

A few respondents also expressed concerns about nonphysician health workers managing combinations with more than two drug components, as this requires specialized skills beyond their current capacity:

If the nonphysician health worker is to use fixed-dose combinations, they should stick to two agents first. If BP control is not achieved, they should refer to a physician. (Cardiologist, Secondary Healthcare Hospital)

(I)t’s to grade the drugs, like the basic drugs, those ones you see them in fixed dose in some places, but not all classes of drugs should be made available to the people at the primary health care or to CHEWs or nurses. They might not be able to handle because they cannot give all the necessary information concerning certain drugs. (Pharmacist Policy Maker, Federal Ministry of Health)

Nonetheless, HCWs expressed satisfaction with the ease of dispensing SPC and the simplicity of documenting and monitoring patient progress using this therapy:

Easy to give, easy to record, *and* easy to monitor the client. (CHEWs, PHC Facility Service Providers Chorused Responses)

#### Ease of Use and Reduced Pill Burden

The simplified single-pill format of SPCs facilitated acceptance by HCWs and was widely perceived as making hypertension management easier:

Before, its polypharmacy we are always dealing with, but now you see combined drugs which we can use. (CHEW, PHC Facility Service Provider)

Key informants also highlighted the reduced pill burden as an important advantage of SPCs:

Fixed dose combination is there to ease and reduce pill burden. (Pharmacist, policy maker, Federal Ministry of Health)

If you are to take three pills for a disease and the three are compounded into one pill, compliance with one pill is usually higher. (Cardiologist, Secondary Healthcare Hospital)

### Implementation (Fidelity)

#### SPC Medication Stockouts

Some HCWs in the FGDs reported occasional SPC stockouts, which disrupted consistent prescribing. During stockouts, HCWs issued prescriptions for patients to purchase BP-lowering medications from private pharmacies. However, some patients were reluctant to do so, choosing instead to wait for restocks at PHCs due to concerns about affordability and the quality of medications from private pharmacies:

The combination for losartan is not available sometimes and when a combination isn’t available, we write for them to buy outside, but they say the one we’re giving them in the PHC is better, so they wait. (CHEW, PHC Facility Service Provider)

#### Difficulty Attributing Side Effects to Specific SPC Component

Participants described challenges in identifying and managing side effects associated with individual SPC components, which in turn affected the continued use of the medication:

Sometimes when you are using fixed-dose combinations, when a patient has a side effect to one of the components, it makes it difficult for you to continue using that drug. (Cardiologist, Secondary Healthcare Hospital)

#### Adequate Supervision and Technical Support

Participants emphasized that routine supervision and a clear understanding of role boundaries were essential for ensuring appropriate medication dispensing by HCWs and adherence to treatment protocols:

If they know their limits and are supervised regularly, we can ensure the right drugs are supplied and used appropriately. (Public Health Physician, Policy Maker, Federal Agency)

### Maintenance

#### HCW-Reported Patient Satisfaction and Motivation to Continue SPC Treatment

HCWs reported that patients expressed satisfaction and enthusiasm for the SPC therapy, which motivated regular clinic attendance and treatment adherence:

They come at least every month with their empty cards, to access the next dose, and they are happy, they are very happy, they don’t even want the drug to stop, or this research to stop. (Nurse, PHC Facility Service Provider)

#### SPC Medication Stockouts and Continuity of Care

The effect of stockouts on consistent prescription as outlined above under fidelity also plays a role in influencing the continuity and sustainability of patient engagement with SPC therapy.

#### Sustaining SPC Supply

Policymakers highlighted the potential of a DRF to sustain the supply of SPCs, including the option of introducing a small, subsidized fee to ensure continued availability at the PHCs. However, some expressed concerns about the implications of transitioning from free medication to a fee-based model:

When they (the implementers) say there is money involved you won’t see them (the patients) again, even if it is N100, you won’t see them again. (CHEW, PHC Facility Service Provider)

## Discussion

Understanding the implementation outcomes on the use of SPC antihypertensive therapy delivered by nonphysician HCWs in PHCs in Nigeria is critical for informing scaling up and sustaining the use of SPC through task-sharing initiatives in Nigeria and similar contexts. The explanatory sequential mixed methods used the RE-AIM QuEST to integrate quantitative findings from a cluster RCT with qualitative insights from HCWs and policymakers. Important barriers and facilitators were identified in this study, including the free availability and ease of use of SPCs, improved HCW capacity through training and supervision, and high patient satisfaction as key enablers, while stockouts, limited SPC formulation options, difficulty managing side effects, concerns about affordability, and the scope of nonphysician prescribing emerged as notable challenges. There is a dearth of studies on the implementation of SPCs from HCWs’ and policymakers’ perspectives in LMICs including Africa, and Nigeria specifically, where the burden of hypertension is high and rapidly rising (14).

### Reach and adoption

HCWs’ acceptability of SPCs for the treatment of hypertension was demonstrated by high levels of reach and adoption in this study. This universal adoption also reflects the feasibility of integrating the use of SPCs into PHCs in similar low-resource settings (10,11,31). The perceived benefits of SPCs and the role of training in facilitating HCWs’ adoption of the use of SPC are similar to the findings by Mbuthia *et al*. in Kenya, which highlighted the role of capacity-building for HCWs in prescribing SPC and providing medication adherence counseling for patients (32). The free provision of medications in both the SPC and comparator groups reduced financial barriers, enabling access even in rural areas where hypertension services have historically been limited (4). Increased attendance may therefore reflect the effect of the zero-cost medication rather than the SPC modality alone. Hence, the feasibility of the task-sharing strategy to improve access to treatment with SPC is complemented by the mitigation of financial barriers for patients. These findings are consistent with findings from similar settings in Africa where task-sharing/shifting initiatives improved access, but cost and geographic barriers remain critical determinants of access to care (32,33).

### Effectiveness

The embedded cluster RCT within the HTN Program found that SPCs were as effective as free-equivalent antihypertensive medications for achieving BP control when prescribed by nonphysician HCWs at Steps 2 and 3 of the treatment protocol (19). This result contrasts with findings from the VERONICA trial in Nigeria and the TRIUMPH trial in Sri Lanka, where initiating therapy with SPCs demonstrated superior effectiveness compared with free-equivalent regimens, with BP control rates of 82% versus 72% and 70% versus 55%, respectively (10,11). This difference may reflect the timing of SPC introduction: in the HTN Program, SPCs were introduced at later treatment steps, whereas VERONICA and TRIUMPH evaluated first-line SPC initiation (10,11). Together, these findings underscore that while SPCs may not always show superior clinical effectiveness when introduced later in the treatment protocol, they offer meaningful implementation advantages, including simplified prescribing and enhanced adherence, which warrant evaluation in real-world primary healthcare settings (6,10,11,19).

Despite this, the 54% BP control rate observed among patients randomized to receive SPCs in the HTN Program represents a notable achievement in the Nigerian context, where national hypertension control rates are estimated at 12%, and global control rates average less than 30% (1,34). In some African countries, rates are reported as low as 7%, highlighting the substantial gap in effective hypertension management across the continent (34,35).

These findings align with evidence from other LMICs demonstrating that task-sharing models combined with simplified treatment protocols can achieve substantial improvements in hypertension control (6,32). High-quality studies have also shown that SPC regimens enhance adherence by reducing pill burden, thereby improving clinical outcomes and facilitating hypertension management in resource-limited settings (33,34).

### Implementation (Fidelity)

The relatively high BP control rate observed in this study suggests that adherence to treatment protocols by HCWs for patients on SPCs likely contributed to their effectiveness. However, the proportions of eligible patients placed on SPCs over the six months (21–37%) highlight gaps in fidelity to consistent SPC prescribing. Despite high levels of reach and adoption, not all eligible patients received SPCs as expected. This suggests that, while SPCs contributed to BP control in over half of the treated patients, broader application could have further improved hypertension outcomes (36,37). Several factors potentially influenced HCWs’ fidelity, including concerns about side effects, patient monitoring practices, availability of medications, and supportive supervision. Although dispensing increased over time, qualitative findings indicate that supply-chain interruptions, intermittent stockouts, and patient-level factors may have limited consistent SPC use, though HCWs did not perceive these factors as significant barriers. These findings align with previous literature on implementation barriers to antihypertensive medication uptake and fidelity (32,38,39,40,41). Higher SPC use among patients in the SPC arm of the RCT near the study end attenuates this concern, which may suggest growing HCW familiarity, improved supply, or adaptation over time.

Reservations from some policymakers and HCWs regarding the ability to identify specific drug components responsible for side effects are consistent with previous research, including from Kenya (32,42). These concerns underscore the importance of equipping HCWs with the skills to monitor and manage adverse effects (43). The HTN Program’s structured training on SPC prescribing may have mitigated some of these knowledge gaps (32). There were also mixed opinions on the scope of nonphysician HCWs’ roles in hypertension management. While many stakeholders expressed optimism about their involvement, some advocated restricting their responsibilities to early-stage hypertension and simple two-drug SPCs. These perceptions align with findings by Adeyemo *et al*. on the perception of physicians on the management of hypertension by nonphysician HCWs in Nigeria (44). Challenges related to side effect management and the limited availability of diverse SPC formulations to address patient variability have also been reported in other LMIC settings (6,32). The role of supportive supervision emerged as a key strategy to strengthen fidelity and ensure adherence to SPC protocols. Similar task-sharing/shifting initiatives in resource-limited settings have successfully leveraged regular supervision to maintain quality of care (43,45,46). Supportive supervision was a core element of the HTN Program’s implementation strategy and likely contributed to its success, including the effective use of SPCs (19,47).

### Maintenance

While SPCs offer clear advantages for hypertension management, sustaining their use in resource-limited PHCs staffed by nonphysician HCWs presents notable challenges. These challenges include limited availability, supply chain disruptions, high cost, and insufficient awareness among healthcare providers, as noted by Salam *et al*. (6). These implementation barriers are consistent with our findings where stockouts and potential high out-of-pocket costs have been reported, especially with purchases from private pharmacies. Although SPCs are often perceived as more expensive than free-equivalent regimens (48,49), evidence suggests that large-scale procurement through governments or insurance schemes can offset costs and render SPCs cost-effective (6,50). These findings related to the affordability of BP-lowering medications, including SPC, are consistent with prior research (51,52,53). The planned introduction of a DRF scheme to mitigate stockouts and sustain supply at a subsidized cost aligns with global and local recommendations and practices (54). Results on the implementation and effectiveness of the use of a DRF scheme in the HTN Program indicate that a DRF can mitigate the challenges associated with stockouts of quality BP-lowering medications and lower subsequent out-of-pocket costs for patients (62). The reported stockouts of SPCs may have been linked to the global supply chain disruption during the COVID-19 pandemic in 2020–2021 (55,56,57). Furthermore, the HTN Program’s efforts to sustain SPC supply could leverage community-based insurance initiatives, such as the Basic Health Care Provision Fund, which directly finances PHCs in Nigeria and provides a platform for scaling up SPC therapies (52,58,59).

### Strengths and limitations

This study complements several studies that have demonstrated the acceptability and advantages of SPCs from the patient’s perspective (50). The study adds to this evidence by providing insights into the nonphysician HCWs’ perspectives on using SPC for the treatment of hypertension. While the sample size was small and thus the results may not be generalizable beyond the Federal Capital Territory of Nigeria, they provide contextual inferences on implementation that could guide in similar contexts, especially with similar primary healthcare structures throughout and beyond Nigeria. This study may have benefited from a comparative qualitative inquiry on the perspective of nonphysician HCW in using the free-equivalent medication; however, baseline assessment of the HTN Program suggested the nonphysician HCWs were more familiar with the use of the free-equivalent medications for treatment of hypertension (24). Although the focus of this study was on the perspective of HCWs, it may have benefited from additional insights from patients. While social desirability bias is a potential concern in qualitative research, its impact may have been mitigated by the perspectives of key informants, including policymakers and regulators. As all medications were provided free of charge during the study, Reach may have been enhanced by the availability of free drugs rather than by the SPC intervention alone. This has important implications for sustainability and scalability, which were partially addressed through the establishment of a DRF at HTN Program sites after the trial concluded, which is designed to provide hypertension medications at low cost.

## Conclusion

Implementation of SPC medications for hypertension treatment by nonphysician HCWs at PHCs in Nigeria has shown promising results. The current study demonstrated high levels of reach and adoption, with all participating facilities agreeing to dispense SPCs, and HCWs expressing satisfaction with the ease of dispensing and monitoring patient progress. SPCs are associated with improved BP control rates, which helps make treatment more accessible and manageable for patients. The findings demonstrate that task-sharing strategies supported by simplified SPC regimens can strengthen hypertension management in resource-limited settings, aligning with global calls to decentralize noncommunicable disease care (60,61). However, challenges such as stockouts and concerns about costs with the transition from free drugs to a DRF system were noted. These findings suggest that SPCs can be effectively integrated into primary healthcare settings in Nigeria, provided that adequate training, supervision, and importantly, a reliable supply of affordable and quality medications are ensured.

## Data Accessibility Statement

The datasets generated and/or analyzed during this study are not publicly available due to ethical restrictions and privacy concerns but are available from the corresponding author upon reasonable request and subject to approval. Quantitative data in this mixed method are part of a cluster RCT that has been published and duly cited in this manuscript.

## Additional File

The additional file for this article can be found as follows:

10.5334/gh.1507.s1 Supplementary File. Supplement 1 to 3.

## References

[1] Odili AN, Chori BS, Danladi B, Nwakile PC, Okoye IC, Abdullah U, et al. Prevalence, awareness, treatment and control of hypertension in Nigeria: data from a nationwide survey 2017. Glob Heart. 2020;15(1):47. DOI: 10.5334/gh.848 32923341 PMC7427662

[2] Burnier M, Egan BM. Adherence in hypertension. Circ Res. 2019;124(7):1124–1140. DOI: 10.1161/CIRCRESAHA.118.313220 30920917

[3] Jobe M, Beye SM, Gaye ND, Ka MM, Perel P, Perkins AD, et al. Hypertension in sub-Saharan Africa: burden, barriers and priorities for improving treatment outcomes. Circ Res. 2025;137(1):106–118. DOI: 10.1161/CIRCRESAHA.124.323889 40536937 PMC12175831

[4] Seedat YK. Why is control of hypertension in sub-Saharan Africa poor? Cardiovasc J Afr. 2015;26(4):193–5. DOI: 10.5830/CVJA-2015-065 26407222 PMC4683286

[5] Du LP, Cheng ZW, Zhang YX, Li Y, Mei D. The impact of fixed-dose combination versus free-equivalent combination therapies on adherence for hypertension: a meta-analysis. J Clin Hypertens. 2018;20(5):902–907. DOI: 10.1111/jch.13272 PMC8030969 29700923

[6] Salam A, Huffman MD, Kanukula R, Prasad EH, Sharma A, Heller DJ, et al. Two-drug fixed-dose combinations of blood pressure-lowering drugs as WHO essential medicines: an overview of efficacy, safety, and cost. J Clin Hypertens. 2020;22(10):1769–1779. DOI: 10.1111/jch.14009 PMC8030031 32815663

[7] Kishore SP, Salam A, Rodgers A, Jaffe MG, Frieden T. Fixed-dose combinations for hypertension. The Lancet. 2018;392(10150):819–820. DOI: 10.1016/S0140-6736(18)31814-2 30215377

[8] Benjamin IJ, Kreutz R, Olsen MH, Schutte AE, Lopez-Jaramillo P, Frieden TR, et al. Fixed-dose combination antihypertensive medications. The Lancet. 2019;394(10199):637–638. DOI: 10.1016/S0140-6736(19)31629-0 31320200

[9] An J, Derington CG, Luong T, Olson KL, King JB, Bress AP, et al. Fixed-dose combination medications for treating hypertension: a review of effectiveness, safety, and challenges. Curr Hypertens Rep. 2020;22:1–19. DOI: 10.1007/s11906-020-01109-2 33052522

[10] Webster R, Salam A, de Silva HA, Selak V, Stepien S, Rajapakse S, et al. Fixed low-dose triple combination antihypertensive medication vs usual care for blood pressure control in patients with mild to moderate hypertension in Sri Lanka: a randomized clinical trial. JAMA. 2018;320(6):566–579. DOI: 10.1001/jama.2018.10359 30120478 PMC6583010

[11] Ojji DB, Salam A, Sani MU, Ogah OS, Schutte AE, Huffman MD, et al. Low-dose triple-pill vs standard-care protocols for Hypertension Treatment in Nigeria: a randomized clinical trial. JAMA. 2024;332(13):1070–1079. DOI: 10.1001/jama.2024.18080 39215620 PMC11366076

[12] Salam A, Atkins ER, Hsu B, Webster R, Patel A, Rodgers A. Efficacy and safety of triple versus dual combination blood pressure-lowering drug therapy: a systematic review and meta-analysis of randomized controlled trials. J Hypertens. 2019;37(8):1567–1573. DOI: 10.1097/HJH.0000000000002089 31058799

[13] Kadiri S, Arogundade F, Arije A, Omotoso A, Onwubere B, Aderibigbe A, et al. Guidelines for the management of hypertension in Nigeria 2020. Tropical Journal of Nephrology. 2020;15(1):65–84.

[14] Ojji DB, Huffman MD. The place of polypill in secondary prevention of stroke. Lancet Glob Health. 2023;11(10):e1488–e1489. DOI: 10.1016/S2214-109X(23)00407-2 37734783 PMC12266284

[15] Callaghan M, Ford N, Schneider H. A systematic review of task-shifting for HIV treatment and care in Africa. Hum Resour Health. 2010;8(1):8. DOI: 10.1186/1478-4491-8-8 20356363 PMC2873343

[16] Rabkin M, El-Sadr WM. Why reinvent the wheel? Leveraging the lessons of HIV scale-up to confront non-communicable diseases. Glob Public Health. 2011;6(3):247–256. DOI: 10.1080/17441692.2011.552068 21390970

[17] Mwai GW, Mburu G, Torpey K, Frost P, Ford N, Seeley J. Role and outcomes of community health workers in HIV care in sub-Saharan Africa: a systematic review. J Int AIDS Soc. 2013;16(1):18586. DOI: 10.7448/IAS.16.1.18586 24029015 PMC3772323

[18] Adebayo MA, Adeniyi BO, Oluwasanu M, Hassan A, Ajuwon GA, Ogbuji QC, et al. Tuberculosis treatment outcomes and associated factors in two states in Nigeria. Trop Med & Int Health. 2020;25(10):1261–1270. DOI: 10.1111/tmi.13467 32677754

[19] Sanuade OA, Ale BM, Baldridge AS, Orji IA, Shedul GL, Ojo TM, et al. Fixed-dose combination therapy-based protocol compared with free pill combination protocol: Results of a cluster randomized trial. J Clin Hypertens. 2023;25(2):127–136. DOI: 10.1111/jch.14632 PMC9903187 36660886

[20] Orji IA, Baldridge AS, Omitiran K, Guo M, Ajisegiri WS, Ojo TM, et al. Capacity and site readiness for hypertension control program implementation in the Federal Capital Territory of Nigeria: a cross-sectional study. BMC Health Serv Res. 2021;21(1). DOI: 10.1186/s12913-021-06320-8 PMC8034094 33836719

[21] Baldridge AS, Aluka-Omitiran K, Orji IA, Shedul GL, Ojo TM, Eze H, et al. Hypertension Treatment in Nigeria (HTN) Program: rationale and design for a type 2 hybrid, effectiveness, and implementation interrupted time series trial. Implement Sci Commun. 2022;3(1):84. DOI: 10.1186/s43058-022-00328-9 35918703 PMC9344662

[22] National Primary Health Care Development Agency. *National Standing Orders*, for *Community Health Officers/Community Health Extension Workers*. 2020; Bill Shakespeare.

[23] Okpetu EI, Abimbola S, Koot J, Kane S. Implementing prevention interventions for non-communicable diseases within the primary health care system in the Federal Capital Territory, Nigeria. Journal Community Med Prim Health Care. 2018;30(1):1–18.

[24] Ojji DB, Baldridge AS, Orji IA, Shedul GL, Ojo TM, Ye J, et al. Characteristics, treatment, and control of hypertension in public primary healthcare centers in Nigeria: baseline results from the Hypertension Treatment in Nigeria Program. J Hypertens. 2022;40(5):888–896. DOI: 10.1097/HJH.0000000000003089 35034080 PMC9081131

[25] World Health Organization. Guideline for the pharmacological treatment of hypertension in adults. World Health Organization; 2021. 34495610

[26] World Health Organization. Hearts: technical package for cardiovascular disease management in primary health care. 2021. World Health Organization; 2021. Accessed May 10, 2025.

[27] Schoonenboom J, Johnson RB. How to construct a mixed methods research design. KZfSS Kölner Zeitschrift für Soziologie und Sozialpsychologie. 2017;69(S2):107–131. DOI: 10.1007/s11577-017-0454-1 28989188 PMC5602001

[28] Forman J, Heisler M, Damschroder LJ, Kaselitz E, Kerr EA. Development and application of the RE-AIM QuEST mixed methods framework for program evaluation. Prev Med Rep. 2017;6:322–328. DOI: 10.1016/j.pmedr.2017.04.002 28451518 PMC5402634

[29] Tong A, Sainsbury P, Craig J. Consolidated criteria for reporting qualitative research (COREQ): a 32-item checklist for interviews and focus groups. Int J Qual Health Care. 2007;19(6):349–57. DOI: 10.1093/intqhc/mzm042 17872937

[30] Dedoose. Dedoose Version 9.0.107, cloud application for managing, analyzing, and presenting qualitative and mixed method research data. 2023 [cited 2025 March 6th]. Available from: https://www.dedoose.com/.

[31] Campbell NRC. A step in the global effort to control hypertension: fixed dose combination antihypertensive drugs. J Clin Hypertens (Greenwich). 2019;21(9):1426–1428. DOI: 10.1111/jch.13683 31441988 PMC8030419

[32] Mbuthia D, Willis R, Gichagua M, Nzinga J, Mugo P, Murphy A. Acceptability of fixed-dose combination treatments for hypertension in Kenya: a qualitative study using the theoretical framework of acceptability. medRxiv. 2024:2024.02.23.24303258. DOI: 10.1101/2024.02.23.24303258 PMC11918355 40100817

[33] Okello S, Muhihi A, Mohamed SF, Ameh S, Ochimana C, Oluwasanu AO, et al. Hypertension prevalence, awareness, treatment, and control and predicted 10-year CVD risk: a cross-sectional study of seven communities in East and West Africa (SevenCEWA). BMC Public Health. 2020;20(1):1706. DOI: 10.1186/s12889-020-09829-5 33187491 PMC7666461

[34] Mills KT, Bundy JD, Kelly TN, Reed JE, Kearney PM, Reynolds K, et al. Global disparities of hypertension prevalence and control. Circulation. 2016;134(6):441–450. DOI: 10.1161/CIRCULATIONAHA.115.018912 27502908 PMC4979614

[35] Sorato MM, Davari M, Kebriaeezadeh A, Sarrafzadegan N, Shibru T, Fatemi B. Reasons for poor blood pressure control in Eastern sub-Saharan Africa: looking into 4P’s (primary care, professional, patient, and public health policy) for improving blood pressure control: a scoping review. BMC Cardiovasc Disord. 2021;21(1):123. DOI: 10.1186/s12872-021-01934-6 33663387 PMC7971125

[36] Brown MT, Bussell JK. Medication adherence: who cares? Mayo Clin Proc. 2011;86(4):304–14. DOI: 10.4065/mcp.2010.0575 21389250 PMC3068890

[37] Kim H, Yoon HJ, Park HS, Cho YK, Nam CW, Han S, et al. Implications of prescribing a fixed-dose combination in clinical cardiology practice: a retrospective observational study using a single medical centre database in Korea. Heart Asia. 2017;9(2):e010885. DOI: 10.1136/heartasia-2017-010885 29467832 PMC5818044

[38] Swanlund SL. Successful cardiovascular medication management processes as perceived by community-dwelling adults over age 74. Appl Nurs Res. 2010;23(1):22–29. DOI: 10.1016/j.apnr.2008.03.005 20122507

[39] Drumond N, van Riet-Nales DA, Karapinar-Çarkit F, Stegemann S. Patients’ appropriateness, acceptability, usability and preferences for pharmaceutical preparations: results from a literature review on clinical evidence. Int J Pharm. 2017;521(1):294–305. DOI: 10.1016/j.ijpharm.2017.02.029 28229945

[40] Osuafor NG, Ukwe CV, Okonta M. Evaluation of availability, price, and affordability of cardiovascular, diabetes, and global medicines in Abuja, Nigeria. PLOS ONE. 2021;16(8):e0255567. DOI: 10.1371/journal.pone.0255567 34383799 PMC8360378

[41] Ndichu ET, Ohiri K, Sekoni O, Makinde O, Schulman K. Evaluating the quality of antihypertensive drugs in Lagos State, Nigeria. PLOS One. 2019;14(2):e0211567. DOI: 10.1371/journal.pone.0211567 30759124 PMC6373917

[42] Essa M, Ross JS, Dhruva SS, Desai NR, Spatz ES, Faridi KF. Utilization of fixed-dose combination treatment for hypertension in Medicare and Medicaid from 2016 to 2020. Circ: Cardiovasc Qual Outcomes. 2024;17(5):e010697. DOI: 10.1161/CIRCOUTCOMES.123.010697 38712553

[43] Joshi R, Alim M, Kengne AP, Jan S, Maulik PK, Peiris D, et al. Task shifting for non-communicable disease management in low and middle income countries—a systematic review. PLOS One. 2014;9(8):e103754. DOI: 10.1371/journal.pone.0103754 25121789 PMC4133198

[44] Adejumo OA, Ogundele OA, Mamven M, Otubogun FM, Junaid OA, Okoye OC, et al. Physicians’ perception of task sharing with non-physician health care workers in the management of uncomplicated hypertension in Nigeria: a mixed method study. PLOS One. 2023;18(9):e0291541. DOI: 10.1371/journal.pone.0291541 37756324 PMC10529560

[45] Adeyemo A, Tayo BO, Luke A, Ogedegbe O, Durazo-Arvizu R, Cooper R. The Nigerian antihypertensive adherence trial: a community-based randomized trial. J Hypertens. 2013;31(1):201–7. DOI: 10.1097/HJH.0b013e32835b0842 23137954 PMC3530610

[46] Morris-Paxton AA, Rheeder P, Ewing R-MG, Woods D. Detection, referral and control of diabetes and hypertension in the rural Eastern Cape Province of South Africa by community health outreach workers in the rural primary healthcare project: health in every hut. Afr J Prim Health Care Fam Med. 2018;10(1):e1–e8. DOI: 10.4102/phcfm.v10i1.1610 PMC5913786 29781685

[47] Shedul GL, Ripiye N, Jamro EL, Orji IA, Shedul GJ, Ugwuneji EN, et al. Supportive supervision visits in a large community hypertension programme in Nigeria: implementation methods and outcomes. BMJ Open Quality. 2025;14(1):e003163. DOI: 10.1136/bmjoq-2024-003163 PMC11934367 40127954

[48] Hong SH, Wang J, Tang J. Dynamic view on affordability of fixed-dose combination antihypertensive drug therapy. Am J Hypertens. 2013;26(7):879–887. DOI: 10.1093/ajh/hpt035 23512697

[49] Bramlage P, Schmidt S, Sims H. Fixed-dose vs free-dose combinations for the management of hypertension-An analysis of 81 958 patients. J Clin Hypertens (Greenwich). 2018;20(4):705–715. DOI: 10.1111/jch.13240 29457348 PMC8031114

[50] Parati G, Kjeldsen S, Coca A, Cushman WC, Wang J. Adherence to single-pill versus free-equivalent combination therapy in hypertension. Hypertension. 2021;77(2):692–705. DOI: 10.1161/HYPERTENSIONAHA.120.15781 33390044

[51] Zhou X, Zhang X, Gu N, Cai W, Feng J. Barriers and facilitators of medication adherence in hypertension patients: a meta-integration of qualitative research. Journal of Patient Experience. 2024;11:23743735241241176. DOI: 10.1177/23743735241241176 38549805 PMC10976505

[52] Harrison MA, Marfo AFA, Opare-Addo MNA, Ankrah DNA, Acheampong F, Nelson F, et al. Anti-hypertensive medication access and affordability and their association with blood pressure control at a teaching hospital in Ghana. Pan Afr Med J. 2021;39:184. DOI: 10.11604/pamj.2021.39.184.27977 34584609 PMC8449564

[53] Edward A, Campbell B, Manase F, Appel LJ. Patient and healthcare provider perspectives on adherence with antihypertensive medications: an exploratory qualitative study in Tanzania. BMC Health Serv Res. 2021;21(1):834. DOI: 10.1186/s12913-021-06858-7 34407820 PMC8371775

[54] Shedul G, Sanuade OA, Ugwuneji EN, Ojo TM, Vijay A, Ponzing P, et al. Stakeholder perspectives on the demand and supply factors driving substandard and falsified blood pressure lowering medications in Nigeria: a qualitative study. BMJ Open. 2022;12(12):e063433. DOI: 10.1136/bmjopen-2022-063433 PMC9791447 36549744

[55] Jifar WW, Geneti GB, Dinssa SD. The Impact of COVID-19 on pharmaceutical shortages and supply disruptions for non-communicable diseases among public hospitals of South West, Oromia, Ethiopia. J Multidiscip Healthc. 2022;15:1933–1943. DOI: 10.2147/JMDH.S377319 36072279 PMC9442910

[56] Badreldin HA, Atallah B. Global drug shortages due to COVID-19: impact on patient care and mitigation strategies. Res Social Adm Pharm. 2021;17(1):1946–1949. DOI: 10.1016/j.sapharm.2020.05.017 32446652 PMC7235598

[57] Faiva E, Hashim HT, Ramadhan MA, Musa SK, Bchara J, Tuama YD, et al. Drug supply shortage in Nigeria during COVID-19: efforts and challenges. J Pharm Policy Pract. 2021;14(1):17. DOI: 10.1186/s40545-021-00302-1 33482871 PMC7820524

[58] Ajisegiri WS, Abimbola S, Tesema AG, Odusanya OO, Peiris D, Joshi R. The organisation of primary health care service delivery for non-communicable diseases in Nigeria: a case-study analysis. PLOS Glob Public Health. 2022;2(7):e0000566. DOI: 10.1371/journal.pgph.0000566 36962373 PMC10021956

[59] Ajisegiri WS, Abimbola S, Tesema AG, Odusanya OO, Ojji DB, Peiris D, et al. Aligning policymaking in decentralized health systems: Evaluation of strategies to prevent and control non-communicable diseases in Nigeria. PLOS Glob Public Health. 2021;1(11):e0000050. DOI: 10.1371/journal.pgph.0000050 36962096 PMC10022121

[60] Klassen SL, Okello E, Ferrer JME, Alizadeh F, Barango P, Chillo P, et al. Decentralization and integration of advanced cardiac care for the world’s poorest billion through the PEN-Plus Strategy for severe chronic non-communicable disease. Glob Heart. 2024;19(1):33. DOI: 10.5334/gh.1313 38549727 PMC10976983

[61] Varghese C, Prem A, Nongkynrih B, Chattu VK, Mikkelsen B. Fourth UNHLM on noncommunicable diseases 2025: an opportunity to bridge the transcending priorities for impact in global south. PLOS Glob Public Health. 2025;5(3):e0004287. DOI: 10.1371/journal.pgph.0004287 40106475 PMC11922195

[62] Shedul GJ, Ugwuneji EN, Jamro EL, Banigbe B, Ponzing P, Okpe I, et al. Design and implementation of a drug revolving fund for hypertension treatment in primary care setting of the Federal Capital Territory, Nigeria. BMJ Global Health. 2025;10(11):e018029. DOI: 10.1136/bmjgh-2024-018029 PMC12636893 41265927

