# Supplementary material for: Implementation of Single-Pill Combination Medication for Hypertension Treatment by Nonphysician Health Care Workers at Primary Healthcare Facilities in Nigeria: An Explanatory Mixed Methods Study
Source: Glob Heart. 2025 Dec 22;20(1):112. doi: 10.5334/gh.1507 (PMC12742375; doi:10.5334/gh.1507)
Supplement: Supplementary File. — Supplement 1 to 3. [file gh-20-1-1507-s1.pdf]

Supplement 1:

COREQ^ (COnsolidated criteria for REporting Qualitative research) Checklist for the Implementation of Single-Pill Combination Medication for Hypertension Treatment by Nonphysician Health Care Workers at Primary Healthcare Facilities in Nigeria: An Explanatory Mixed Methods Study

Item No.	Guide Questions / Description	Response	Reported on Page No.
Domain 1: Research team and reflexivity			
Personal Characteristics		Public health professionals<br>Familiarity with the study context	5
1. Interviewer/facilitator	Which author conducted the interview or focus group?	EIO	5
2. Credentials	What were the researcher's credentials? E.g., PhD, MD	MBBS/MD: EIO, BMA, GLS, NRR, IAO, LRH, DBO, MDH<br>MPH/MS: EIO, CO, BMA, RCBO ASB, GLS, NRR, IAO, LRH, MDH<br>PharmD: GJS<br>PhD/DrPH: CO, RCBO, ASB, IAO, DBO	N/A
3. Occupation	What was their occupation at the time of the study?	Research fellows<br>Public health physician<br>Pharmacist<br>Primary care physician<br>Cardiologist<br>Sociologist<br>Biostatistician	1, 5
4. Gender	Was the researcher male or female?	Male and Female	N/A
5. Experience and training	What experience or training did the researcher have?	Qualitative research methods<br>Implementation research<br>Mixed method research	5
Relationship with participants			
6. Relationship established	Was a relationship established prior to study commencement?	Yes	5
7. Participant knowledge of the interviewer	What did the participants know about the researcher?	Some of the participants are familiar with some of the researchers from training, supervision and stakeholders' engagement	N/A
8. Interviewer characteristics	What characteristics were reported about the interviewer/facilitator?	Training in qualitative research and familiarity with the context	5
Domain 2: Study design			
Theoretical framework		RE-AIM/RE-AIM QuEST	4, 5
9. Methodological orientation and theory	What methodological orientation underpinned the study?	Explanatory sequential mixed method (methods section)	4
Participant selection			

10. Sampling	How were participants selected?	Purposively	5
11. Method of approach	How were participants approached?	Phone calls	5
12. Sample size	How many participants were in the study?	29 in the qualitative sub-study	5
13. Non-participation	How many people refused to participate or dropped out? Reasons?	None	N/A
Setting			
14. Setting of data collection	Where was the data collected?	Mutually agreed suitable location	5
15. Presence of non-participants	Was anyone else present besides the participants and researchers?	No	N/A
16. Description of sample	What are the important characteristics of the sample?	Nonphysician health care workers, policy makers, health program managers and health care specialists	Table 2
Data collection			
17. Interview guide	Were questions, prompts, guides provided by the authors? Was it pilot tested?	Yes	5, supplements 2,3
18. Repeat interviews	Were repeat interviews carried out? If yes, how many?	No	5
19. Audio/visual recording	Did the research use audio or visual recording to collect the data?	Yes	5
20. Field notes	Were field notes made during and/or after the interview or focus group?	During	5
21. Duration	What was the duration of the interviews or focus group?	Interviews: 30 minutes, focus groups: 45 minutes	5
22. Data saturation	Was data saturation discussed?	Yes	5
23. Transcripts returned	Were transcripts returned to participants for comment and/or correction?	No	5
Domain 3: Analysis and findings			
Data analysis			
24. Number of data coders	How many data coders were involved in the analysis?	2 coders	5
25. Description of the coding tree	Did authors provide a description of the coding tree?	No	
26. Derivation of themes	Were themes identified in advance or derived from the data?	Abductive- preidentified and derived	5
27. Software	What software, if applicable, was used to manage the data?	Dedoose software (v9.2.22, Los Angeles, CA: Sociocultural Research Consultants, LLC)	5
28. Participant checking	Did participants provide feedback on the findings?	No	N/A
Reporting			

29. Quotations presented	Were participant quotations presented to illustrate the themes/findings?	Yes	7-9
30. Data and findings consistent	Was there consistency between the data presented and the findings?	Yes	6-9
31. Clarity of major themes	Were major themes clearly presented in the findings?	Yes	6-9
32. Clarity of minor themes	Is there a description of diverse cases or discussion of minor themes?	Yes	6-9

^ Tong, A., P. Sainsbury, and J. Craig, Consolidated criteria for reporting qualitative research (COREQ): a 32-item checklist for interviews and focus groups. Int J Qual Health Care, 2007. 19(6): p. 349-57.

Supplement 2

Hypertension Treatment in Nigeria (HTN)
Cardiovascular Research Unit, University of Abuja Teaching Hospital, Gwagwalada, Abuja, Nigeria

Study Assessing Nonphysician Health Care Workers Use of Fixed Dose Combination Medication for Hypertension Treatment in Primary Health Care Facilities in Nigeria

DISCUSSION GUIDE –HEALTH CARE WORKERS (Community Health Extension Workers and Nurses)

Introduction and consent: Participants were welcomed, and the purpose of the discussion was explained. Confidentiality and voluntary participation were emphasized, and informed consent was obtained from each participant prior to the start of the session.

Discussion questions

1. What can you say about the problem of hypertension in your community?
2. What is your experience with managing hypertensive patients in your Primary Health Care (PHC) facility?
3. What is your experience using fixed-dose combination (FDC) medications in managing any disease condition?
4. What types of medications have you used in managing hypertension? Which do you prefer, and why?
5. Have you been introduced to the use of FDCs for managing hypertension?
6. When were you introduced to FDCs for hypertension, and how long have you used them?
7. How would you describe your experience using FDCs for hypertension management?
8. What are your thoughts on the use of FDCs for treating hypertension?
9. Do you have any objections or reservations about using FDCs for hypertension treatment? Please explain.
10. Have you experienced any challenges in using FDCs for managing hypertension?
11. Do you think FDCs are beneficial for your patients? Why or why not?
12. Would you like to continue using FDCs for hypertension management? Why or why not?
13. Are there other medication options you would recommend for the treatment of hypertension? Why?
14. Do you have any additional thoughts, opinions, or recommendations?

THANK YOU ALL FOR YOU TIME

Supplement 3

Hypertension Treatment in Nigeria (HTN)
Cardiovascular Research Unit, University of Abuja Teaching Hospital, Gwagwalada, Abuja, Nigeria
Study Assessing Nonphysician Health Care Workers Use of Fixed Dose Combination Medication for Hypertension Treatment in Primary Health Care Facilities in Nigeria

INTERVIEW GUIDE- POLICY STAKEHOLDERS (healthcare managers and regulators)

Introduction and consent: Participants were welcomed, and the purpose of the discussion was explained. Confidentiality and voluntary participation were emphasized, and informed consent was obtained from each participant prior to the start of the session.

Interview Questions

1. What do you think about the burden of hypertension in Nigeria?

2. What are your thoughts on providing access to treatment for more people?

3. What is your understanding of current policies on the management of hypertension by nonphysicians (e.g., nurses, community health extension workers) in Nigeria?

4. What are your thoughts or experiences regarding the use of fixed-dose combination (FDC) medications for treating any disease?

5. What is your opinion about the use of FDCs in the treatment of hypertension?

6. What do you think about CHEWs, nurses and other nonphysicians being involved in managing hypertension in PHCs?

7. What are your thoughts on the use of FDCs by nonphysician health workers in PHCs for hypertension treatment?

8. Do you have any additional thoughts, opinions, or recommendations?

THANK YOU FOR YOU TIME
